# Development of Wheel Pressure Control Algorithm for Electronic Stability Control (ESC) System of Commercial Trucks

**DOI:** 10.3390/s18072317

**Published:** 2018-07-17

**Authors:** Minjun Seo, Changhee Yoo, Sang-Shin Park, Kanghyun Nam

**Affiliations:** 1 School of Mechanical Engineering, Yeungnam University, 280 Daehak-ro, Gyeongsan 38541, Korea; mjseo@hanhoinc.com (M.S.); pss@ynu.ac.kr (S.-S.P.); 2 Commercial Development Department, Sangsin Brake Co., Ltd., 90, Techno Jungang-Daero, Yuga-Eup, Dalseong-Gun, Daegu 43023, Korea; yooch@sangsin.com

**Keywords:** electronic stability control, commercial truck, wheel pressure control, solenoid valve

## Abstract

This paper presents a wheel cylinder pressure control algorithm for application to the vehicle electronic stability control (ESC) systems for commercial trucks. An ESC system is an active system that improves the driving stability by distributing the appropriate braking pressure to each wheel, which is an essential system for safe driving. It is important that the ESC system, through proper braking pressure supply, delivers the correct pressure under control. However, to reduce the cost involved, commercial trucks use a solenoid valve of the on/off-type, rather than a proportional valve that has good pressure control capability. The performance of a proposed wheel pressure control system based on an on/off solenoid valve control was verified by means of experiments conducted using the wheel pressure control algorithm presented in this paper.

## 1. Introduction

Serious vehicle accidents causing large-scale damage tend to occur because of the involvement of trucks that are heavier than typical passenger vehicles. Heavy-duty commercial trucks require 40% longer braking distances than relatively lightweight vehicles, and thus are more likely to be involved in fatal accidents [1,2,3]. Accordingly, studies on control systems related to driving stability have dealt with commercial trucks as well as passenger vehicles. An electronic stability control (ESC) system stabilizes the movement of a vehicle by being activated when a driver loses his/her control of the vehicle, and the difference between the calculation of the yaw rate in a high-level electronic controller unit (ECU) and the real yaw rate exceeds a certain threshold [4,5,6]. The effectiveness of ESC systems has been verified statistically using the U.S. National Highway Traffic Safety Administration (NHTSA) vehicle crash data, which shows that ESC systems can reduce passenger vehicle crashes by 34% and sport utility vehicle (SUV) crashes by 59%, and can significantly reduce rollover accidents [7]. An anti-lock brake system (ABS) is needed to implement an ESC system in a vehicle. The ABS system controls the slip between the tires and the ground, and maintains the maximum grip/friction between them by considering the most efficient slip ratio and the relationship between the driving forces and the slip ratio [8]. Normally, because an ABS system is based on slip control, it calculates a slip ratio from the difference between the angular velocity of the wheels and the velocity of a vehicle. The slip can also be controlled in other ways to provide braking force to the wheels, as follows: the slip ratio can be estimated without using a velocity sensor [9], the maximum driving force can be obtained by estimating driving forces without slip control [10], and the maximum friction of the tires can also be used [11]. Consequently, an ABS system needs to be used to allocate the optimum braking force to each wheel so that the yaw rate controlled by the ESC system can be satisfied. The ESC and ABS systems of a truck provide and distribute brake pressure to each wheel using control values based on the dynamic properties of the truck. Accordingly, the pressure transmitted to the brakes must be controlled to ensure the accuracy of the brake pressure. This paper presents a wheel pressure control algorithm that transmits accurate pressure to each wheel through the dynamic control of solenoid valves. The proposed algorithm is intended for use in the ESC systems of trucks. The pressure control method proposed in the literature [12,13] was applied to the ABS system of a truck, which transmits the pressure provided by the air reservoir to a wheel cylinder. However, this method exhibited a delay problem. The pressure control method described by Kim and Jang [14] employed the same type of solenoid valve as the actuator used in the experiment in this study, and estimated the pressure using pulse-width modulation (PWM) signals applied to the solenoid valve. The proportional valves typically perform well in precisely controlling the target pressure transmitted from a high-level ECU of a truck. However, when a proportional valve is used as an actuator, the unit cost rises. For this reason, on/off-type solenoid valves are widely used. When such a valve is used, the pressure is switched on and off and the operations of complete opening and closing are performed consecutively. Accordingly, the air pressure applied has nonlinear characteristics, and the consecutive opening and closing of the valve exhibits a stepped response. This study proposes a pressure control algorithm that uses an on/off-type solenoid valve and is applied to the ESC system of a commercial truck. The proposed algorithm configures individual controllers by considering the characteristics of pressure supply and exhaust on the basis of the pneumatic response, as described in Section 4.2. Because the nonlinear characteristics vary depending on the supply pressure, the valve operation conditions are designed as a function of the pressure level, which improves the accuracy of the target pressure estimation. In addition, each of the controllers, which are classified according to their response characteristics, operates in accordance with the gradient of the corresponding target pressure, as described in Section 4.3. Lastly, the difference between the target pressure and the feedback of the pressure sensor is controlled based on bang–bang control for the on/off-type valves [15]. The bang–bang control is modulated based on the threshold for errors, and transmits pressure to the valve for three operational states. The performance of the proposed pressure control algorithm was verified by means of the wheel cylinder pressure control experiment and the pressure control results described in Section 5.

## 2. Overview of Pneumatic ESC System

**Figure 1:**
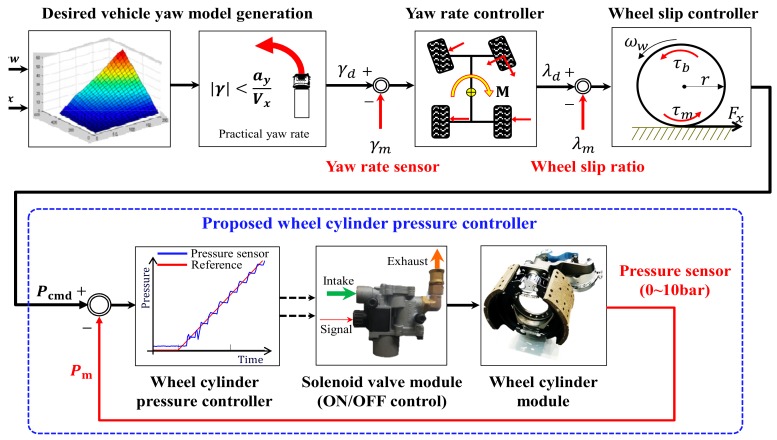
Schematic illustration of a commercial truck’s electronic stability control (ESC) system.

An electronic stability control (ESC) system enhances driving safety by intervening in unintended states such as understeer and oversteer. An ESC system can prevent a vehicle from deviating from its driving course when cornering on a low-friction road surface or during excessive handling [4,5]. Figure 1 presents a schematic diagram of the algorithm of an ESC system. When an ESC system is activated, the desired yaw rates for the driver’s handling angle and the vehicle velocity are calculated, as shown in Formula (1) [4]:

(1) $$\lambda_{d}=\frac{V_{x}}{L+\frac{mV_{x}{}^{2}(l_{r}C_{r}-l_{f}C_{f})}{2C_{f}C_{r}L}}\cdot \delta_{cmd}$$

where $\lambda_{d}$ is the target yaw rate; $V_{x}$ is the velocity of a vehicle in the motion direction; $\delta_{cmd}$ is the steering angle, which depends on the driver’s handling; L is the wheel base; m is the vehicle weight; *l_f_* and *l_r_* are the distance from the center of gravity to the front and rear axles, respectively; and *C_f_* and *C_r_* are the cornering stiffnesses of the front and rear wheels, respectively. However, because $\lambda_{d}$ in Formula (1) is an ideal value, it is modified in Formula (2) by the lateral acceleration $a_{y}$ and velocity $V_{x}$ of the vehicle.

(2) $$\left|\lambda_{d}\right|<\frac{a_{y}}{V_{x}}$$

Accordingly, the control of the yaw rate in rad/sec uses the modified $\lambda_{d}$ in Formula (2) as the target value. A vehicle cannot accurately follow the target $\lambda_{d}$ if its four wheels do not generate appropriate braking forces for the driving conditions. To produce an appropriate braking force, the slip ratio of each wheel is estimated [9] for the purpose of the control of the braking force, and the braking force is provided to each wheel to satisfy the braking torques allocated. The type of commercial truck considered in this study uses a pneumatic brake system. The target pressure allocated to each wheel is transmitted through an adequate brake pressure that is obtained by individual pressure control, as shown in Figure 1.

## 3. Brake System of a Commercial Truck

### 3.1. Brake System of a Commercial Truck

**Figure 2:**
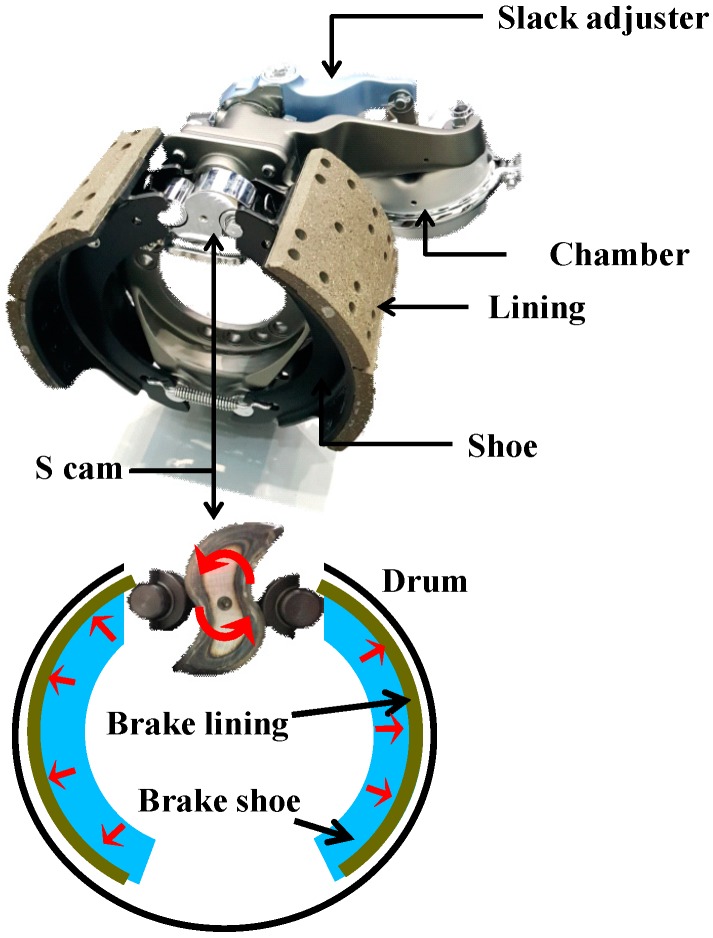
Illustration of a pneumatic drum brake system.

Figure 2 illustrates the structure of the conventional pneumatic drum brake system used in commercial trucks. When the air compressor supplies pressure to the chamber, the drum brake responds with vertical piston movement through the link connected to the slack adjuster. The vertical movement transmitted to the end of the slack adjuster becomes the rotational movement around the axis linked to the S cam. When the S cam begins to rotate, it simultaneously pushes out the brake shoe at both ends, as shown in Figure 2, and the brake lining along the brake shoe creates friction with the brake drum, thereby generating a braking force.

### 3.2. Description of the Pneumatic Circuit for a Commercial Truck’s Brake System

**Figure 3:**
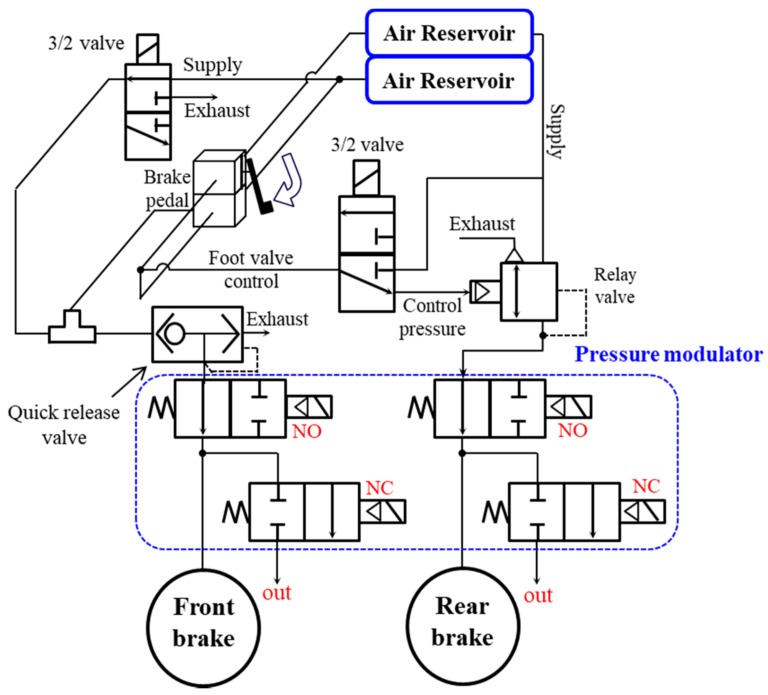
Pneumatic circuit of a commercial truck’s brake system.

In the case of a pneumatic brake, the time required to transport fluid to the wheel cylinder may vary depending on the state of the tube, which is the air passage. Based on Kienhofer and Cebon [12], a quick release valve or relay valve is added between the reservoir and each wheel, and pressure is transmitted to this valve in accordance with the driver’s brake pedaling. Figure 3 illustrates the pneumatic circuit of the brake system of a commercial truck. As Figure 3 shows, a quick-release valve is added to the front wheel, a relay valve or two 3/2 valves are added to the rear wheel, and pressure modulator valves are installed in each of the front and rear wheels to implement the ESC system function. The two 3/2 valves are directly concerned with active braking pressure modulation (e.g., called active braking valve [ABV] and active traction control valve [ATCV], respectively). In accordance with the signals from the high-level ECU, the ABV valve provides brake pressure to the front wheel, and the ATCV supplies brake pressure to the rear wheel. The pressure modulator valves control the air supply and exhaust for each wheel cylinder.

## 4. Wheel Pressure Control Based on Pressure Sensor Feedback

Figure 3 illustrates the pneumatic control of the brake system for commercial trucks. As is clear from the figure, pressure is transmitted from the reservoir tank to the wheel cylinders attached to the wheels through the front and rear pressure modulator valves. The amount of pressure supplied is adjusted by the pressure modulator valves, depending on the driving conditions. This section describes the pressure control achieved using the pressure modulator valves, shown in Figure 3.

### 4.1. Operating Principle of Pressure Modulation Valves

**Figure 4:**
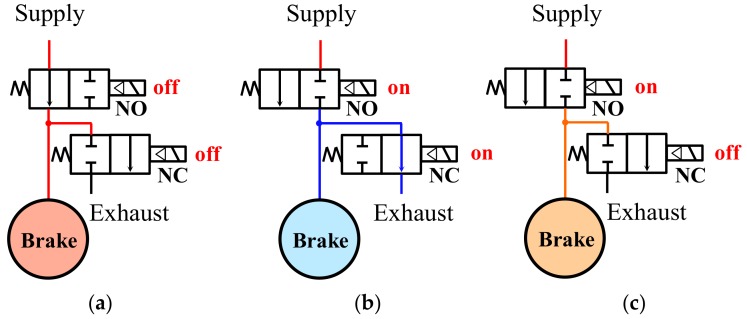
Pressure modulation modes of the (**a**) apply mode; (**b**) dump mode; and (**c**) hold mode.

A pressure modulator valve is composed internally of normally open (NO) and normally closed (NC) valves. The NO valves stay open to allow pressure supply when the power is off and close to interrupt the supply when the power is on. The NC valves close to prevent air exhaust when the power is off and are open to exhaust air from the cylinder when the power is on. Figure 4 illustrates the functioning of the NO and NC valves. The functioning of the valves is divided into three states, illustrated in Figure 4a–c. Pressure control is applied to the pressure control logic in the operational states, shown in Figure 4.

Under the assumption that pressure is not supplied continuously to the supply port, (a) while power is not applied to either the NO or NC valves, pressure is supplied to the wheel cylinder without being exhausted; (b) while power is applied only to the NO valve and the pressure supply is interrupted, pressure is exhausted from the wheel cylinder; or (c) while power is applied only to the NO valve and the pressure inside the wheel cylinder has an appropriate value, the internal pressure of the cylinder is maintained. These operational states are referred to hereinafter as (a) ‘apply mode’, (b) ‘dump mode’, and (c) ‘hold mode’, respectively.

### 4.2. Dynamic Characteristics of a Pneumatic Brake System

**Figure 5:**
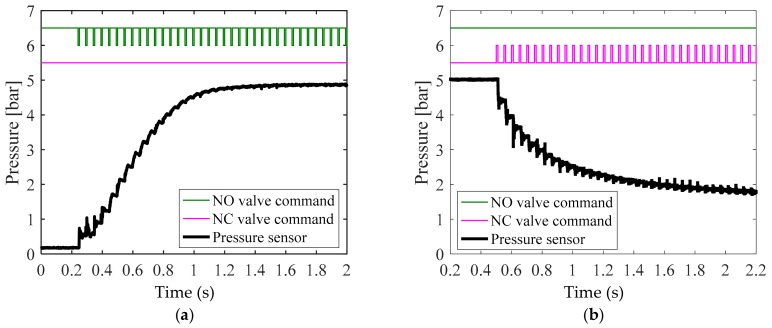
Experimental results for pneumatic valve’s response tests for the (**a**) apply mode test with 20% pulse-width modulation (PWM) duty ratio, and (**b**) dump mode test with 20% PWM duty ratio.

**Figure 6:**
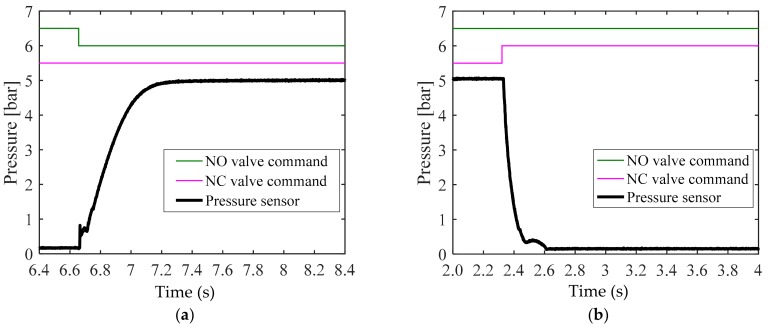
Experimental results for pneumatic valve’s response tests for the (**a**) apply mode test with 100% PWM duty ratio, and (**b**) dump mode test with 100% PWM duty ratio.

The response to the pressure transmitted from the reservoir tank to the wheel cylinder must be tested to design a control algorithm. We conducted an experiment to measure the pneumatic response with the goal of identifying the supply and exhaust characteristics of pressure. For the operation of the solenoid valve, the PWM signal duty ratio was set to 20% and 100%. Figure 5 and Figure 6 present the experimental results for each condition. The supply and exhaust characteristics for each valve operational condition were analyzed as follows. 

Figure 5a illustrates the apply mode. It took 950 ms to achieve the supply pressure of 5 bar. While the pressure was provided, a discontinuous response characteristic due to dead volume was observed at atmospheric pressure. Figure 5b corresponds to the dump mode. It took over 1000 ms to decrease the supply pressure of 5 bar to the atmospheric pressure. It is shown that the response became slower as the supply pressure approached the atmospheric pressure.

Figure 6 illustrates the experimental results obtained by applying a 100% PWM signal duty cycle, which was the maximum operation condition of the valve. Like the data shown in Figure 5, the data in Figure 6 were obtained from an experiment conducted in the (a) apply mode and (b) dump mode. Both (a) and (b) show that a shorter time was required to reach the supply pressure or the atmospheric pressure when applying a 20% duty cycle, than in the case illustrated in Figure 5. In particular, (b) displays the fast response when the pressure was not close to the atmospheric pressure, but as early as near 2 bar, when the supply pressure of 5 bar was exhausted. Consequently, the apply mode exhibited a simple slowdown with a decrease in duty, while a slowdown in the dump mode was exhibited in a specific pressure range when the duty decreased. The analysis of the response characteristics observed in this experiment require a separate control of the apply and dump modes in designing a pressure control algorithm.

### 4.3. Design of a Wheel Cylinder Pressure Controller

**Figure 7:**
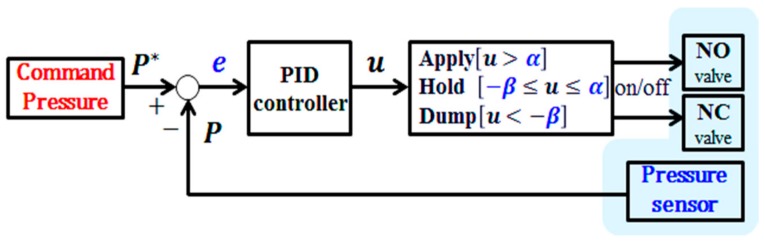
Conventional pressure control method with a simple switching condition.

Figure 7 shows the conventional pressure control algorithm applicable to the brake systems of commercial trucks. As a pressure feedback control algorithm utilizing bang–bang control [15], it outputs a control input value from the difference between a pressure command and a feedback value, through a proportional–integral–derivative (PID) controller. The valves operate in either apply mode or dump mode, depending on the range of set limits of the bang–bang controller. When the control input value is within the set range, the valves operate in hold mode. However, the pressure control algorithm, shown in Figure 7, does not consider the pneumatic response, described in Section 4.2. The pneumatic brake system for commercial trucks has different responses in the pressure supply and exhaust ranges. Accordingly, the algorithm shown in Figure 7 is very likely to generate unnecessary valve operations, and because it uses a large amount of air simultaneously, a larger reservoir tank is needed [8].

**Figure 8:**
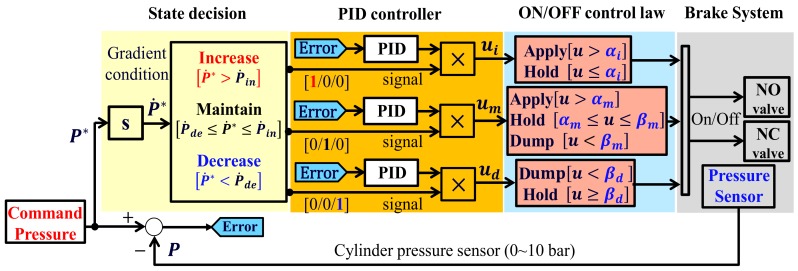
Proposed pressure control method with conditional operating functions.

Based on the pneumatic response described in Section 4.2, the model characteristics of the pressure supply and exhaust were identified experimentally. Accordingly, the architecture of our control algorithm reflected the response characteristics of each model in the design of a controller. For individual pressure control as a function of the pneumatic response, the conditions were classified into three modes in this study—increase, maintain, and decrease—on the basis of the gradient of commands, as shown in the signal decision process illustrated in Figure 8. As shown in Formula (3), when the gradient of command is greater than ${\dot{P}}_{in}$ (defined as the pressure gradient threshold during increase), the signal for the increase mode is the output; when the gradient is less than ${\dot{P}}_{de}$ (defined as the pressure gradient threshold during decrease), the signal for the decrease mode is the output; and when the gradient is between ${\dot{P}}_{in}$ and ${\dot{P}}_{de}$, the signal for the maintain model is the output. ${\dot{P}}_{in}$ and ${\dot{P}}_{de}$ are selected by considering the pressure variation of samples that operate in the apply and dump modes under the minimum duty cycle of 20% for the maintain mode of the pressure modulator valve, shown in Figure 5. Accordingly, different model characteristics are reflected, depending on whether pressure is supplied or exhausted, and while pressure is maintained, unnecessary oscillation due to the on/off control is minimized. The signal outputs obtained under the three conditions are used to judge whether each controller is individually designed according to condition outputs or not.

(3) $$\{\begin{array}{ll} {\dot{P}}^{*}>{\dot{P}}_{in} & :\mathrm{Increase}\\ {\dot{P}}_{de}\leq {\dot{P}}^{*}\leq {\dot{P}}_{in} & :\mathrm{Maintain}\\ {\dot{P}}^{*}<{\dot{P}}_{de} & :\mathrm{Decrease} \end{array}$$

In the pressure control algorithm illustrated in Figure 8, the signals that are output according to the different control modes are transmitted to the bang–bang controller, which determines the operation conditions of the valves by means of the three control inputs ($u_{i},u_{m},u_{d}$), through individually designed controllers. If the control input is $u_{i}$, the gradient is greater than ${\dot{P}}_{in}$. When a valve operating in the apply mode exceeds the limit $\alpha_{i}$ (defined as a control threshold gain), the pressure supply is interrupted by the hold mode. The valve then operates again in the apply mode. If the control input is $u_{d}$, when a valve operating in the dump mode exceeds the limit $\beta_{d}$ (defined as a control threshold gain), the valve begins to operate in the hold mode. If the control input $u_{m}$ is transmitted in a section with a gentle gradient, the valve operates in the apply, hold, and dump modes. As shown in Figure 6, when a controller operates in a section with a drastic change of pressure, an on/off-type valve generates unnecessary oscillation. For this reason, the apply, hold, and dump modes were designed for the limits $\alpha_{m}$ (defined as a control threshold gain) and $\beta_{m}$ (defined as a control threshold gain) to maintain a stable steady state.

(4) $$\{\begin{array}{cc} P^{*}>0.5P^{*} & \Rightarrow \;20\%\;\mathrm{duty}\\ P^{*}<0.3P^{*} & \Rightarrow \;40\%\;\mathrm{duty} \end{array}$$

In addition, the exhaust response characteristics, illustrated in Figure 6b, exhibit an earlier change in pressure that varied depending on the pressure range. The design of the dump mode, illustrated in Figure 8, sets a variable duty mode, as described in Formula (4). In that formula, the operation time of the valve is suggested to be controlled, as follows. In the pressure range above $0.5P^{*}$, which is near the supply pressure, the duty needs to be reduced because of the drastic decrease of pressure, thereby reducing the displacement. On the other hand, in the pressure range below $0.3P^{*}$, which approaches the atmospheric pressure, as the decrease in pressure is small, the duty needs to increase. No individual duty can be set for the range between $0.5P^{*}$ and $0.3P^{*}$, but the duty is varied continuously by the feedback control cycle of 10 ms.

## 5. Experimental Verification

**Figure 9:**
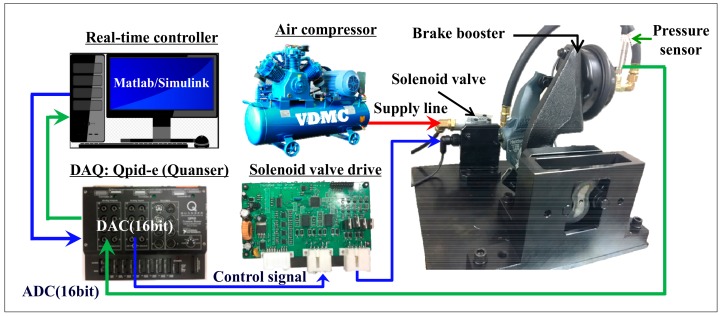
Schematic illustration of experimental setup.

In the pressure control experiment, Matlab/Simulink software was used to confirm the pressure control logic design and real-time control. A DAQ (Quanser qpid-e board) converted an analog signal input to a PWM signal in the solenoid valve PWM drive, and transferred it to the solenoid valves as shown in Figure 9. The components of the pneumatic brake system for real commercial trucks, which include solenoid valves, were provided by Sangsin Brake Co., Ltd. (Daegu, Korea). As shown in Figure 3, the pressure control valve, which is directly connected to the supply line of the air compressor, transmits the pressure from the compressor to the chamber through its on/off control. The slack adjuster modifies the direction of movement to transfer the pressure from the S cam, as braking force. The experimental setup extends as far as the S cam, except for the brake lining and the brake shoe, in Figure 2. The control experiment applied a control cycle of 10 ms on the basis of the high-level ECU used in commercial trucks.

**Figure 10:**
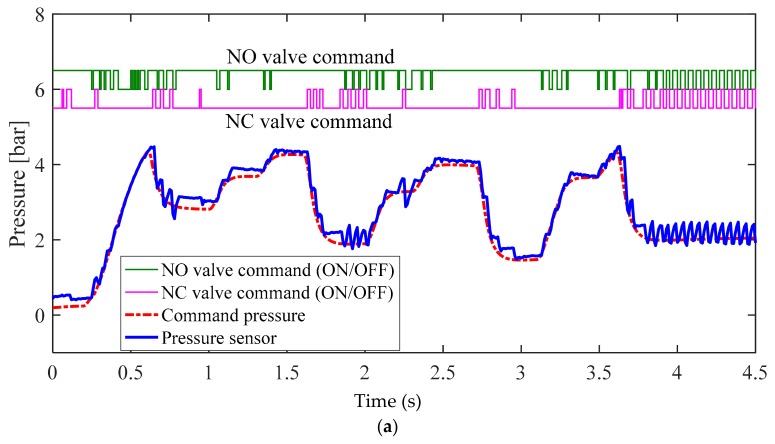

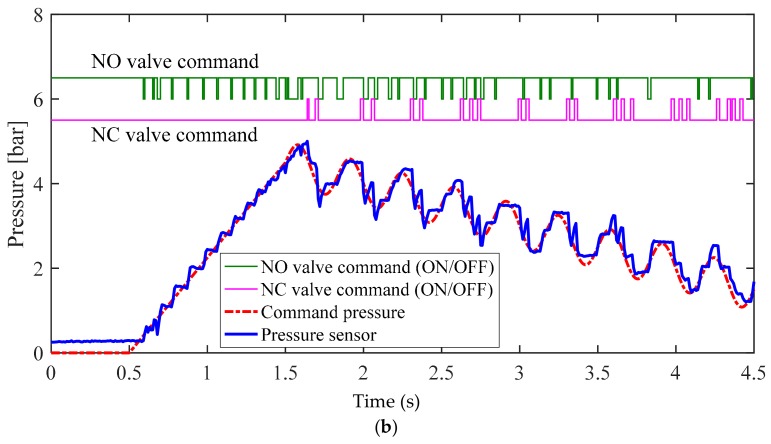
Experimental results for conventional pressure control method in the (**a**) normal braking case and the (**b**) anti-lock brake system (ABS) control case.

**Figure 11:**
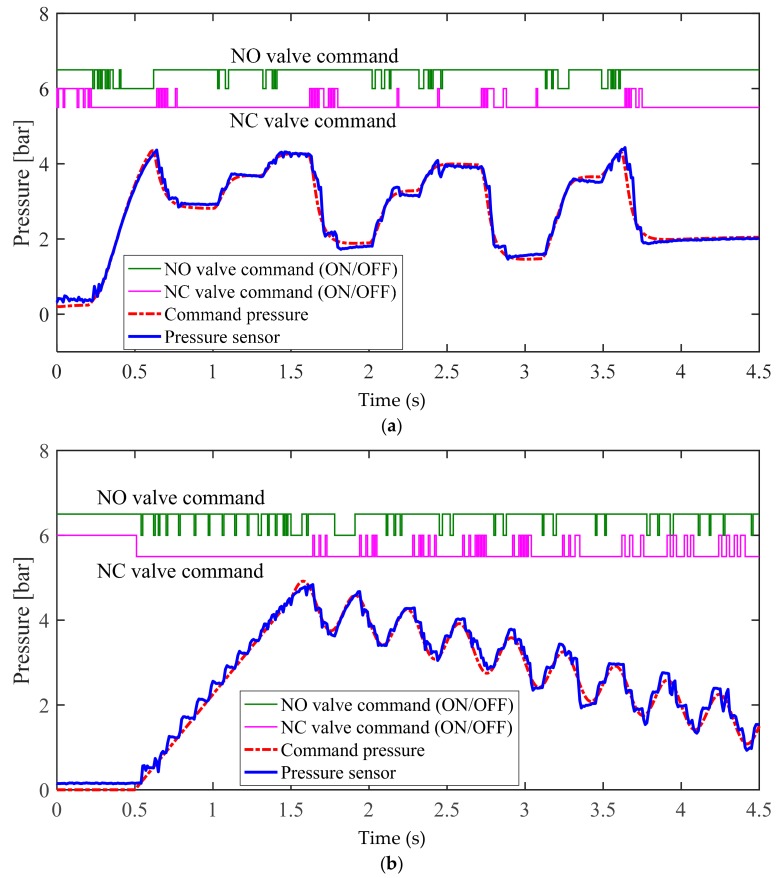
Experimental results for proposed pressure control method in the (**a**) normal braking case and the (**b**) ABS control case.

**Figure 12:**
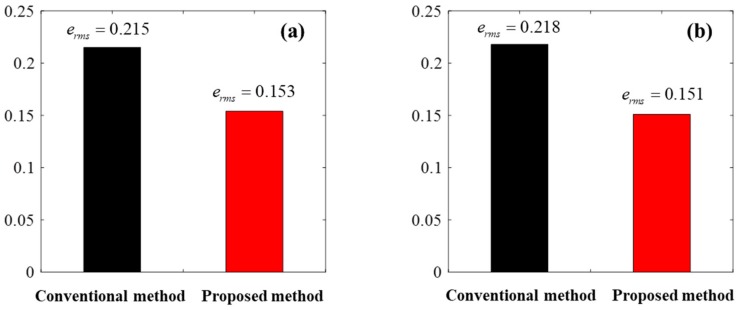
Quantitative comparison (root mean square error) in the (**a**) normal braking case and the (**b**) ABS control case.

Figure 10 illustrates the experimental results for the conventional bang–bang control algorithm, as shown in Figure 7. Figure 11 illustrates the experimental results for the proposed bang–bang control algorithm, as presented in Figure 8, which is based on an individual control strategy. In Figure 10a and Figure 11a, the pressure commands were generated from the simulated irregular patterns of wheel pressure in ABS situations. When the command pressure changes from the decrease or increase state to the maintain state, the steady-state error decreases because of the operational conditions of the controller and valve, which correspond to the maintain state, and the oscillation is removed at the same time. Figure 10b and Figure 11b show the experimental results that verify the transient state in the range between the supply pressure and the atmospheric pressure. When the results in Figure 10 and Figure 11 were compared, it was found that the overall tracking performance for the commands and the reduction in oscillation were improved by applying the proposed control algorithms, which are achieved by individual control for different model characteristics in the apply and dump modes of the internal pressure of the cylinder, which was based on the pneumatic response characteristics. When duty was variably applied in each pressure range, the tracking performance was improved in the dump mode, in which the variation in pressure was relatively larger. The root mean square (rms) values for the control errors obtained from the two experimental cases were calculated and compared to assess the effectiveness of the proposed method quantitatively. Figure 12 shows a 30% improvement in rms error when the proposed method is applied. In the experiments, the tuning variables related to valve control are listed as follows: ${\dot{P}}_{in}=2.5$, ${\dot{P}}_{de}=-5$, $\alpha_{i}=0$, $\alpha_{m}=0.5$, $\beta_{m}=-0.25$, and $\beta_{d}=-0.1$. In summary, the experimental results shown in Figure 10 and Figure 11 were obtained by applying the bang–bang control of the valve operation to the different control inputs under the three gradient conditions of the proposed pressure control algorithm. Because the actuator had no control option other than on/off control, unavoidable control errors occurred. 

## 6. Conclusions

This paper proposes a wheel pressure control algorithm for ESC systems for commercial trucks. An experiment was conducted using an on/off-type pressure control valve as the pressure control actuator for a commercial truck. As the pneumatic brake system exhibited clearly nonlinear characteristics of air pressure and slow responsiveness, the conventional pressure control method, shown in Figure 7, could not achieve the desired reference tracking performance. The proposed wheel pressure control method is to design individual pressure controllers based on the valves’ response characteristics. The performance of the proposed control algorithm was verified by experimental data and discussed. As the air pressure is controlled based on the on/off control of the solenoid valves, slight vibration and noise are inevitably generated. However, the vibration level is greatly improved by applying the proposed control method that variably generates the control amount, reflecting the response characteristics in the supply and exhaust modes. In the next study, we will propose an estimation method, without using a pressure sensor for further study, to achieve the same pressure control performance.
